# Comparison of methods for detoxification of spruce hydrolysate for bacterial cellulose production

**DOI:** 10.1186/1475-2859-12-93

**Published:** 2013-10-12

**Authors:** Xiang Guo, Adnan Cavka, Leif J Jönsson, Feng Hong

**Affiliations:** 1 China-Sweden Associated Research Laboratory in Industrial Biotechnology, College of Chemistry, Chemical Engineering and Biotechnology, Donghua University, Shanghai 201620, China; 2 Group of Microbiological Engineering and Industrial Biotechnology, College of Chemistry, Chemical Engineering and Biotechnology, Donghua University, Shanghai 201620, China; 3 Department of Chemistry, Umeå University, Umeå SE-901 87, Sweden; 4 Industrial Graduate School, Umeå University, Umeå SE-901 87, Sweden

**Keywords:** Bacterial cellulose, *Gluconacetobacter xylinus*, Norway spruce hydrolysate, Detoxification methods

## Abstract

**Background:**

Bacterial cellulose (BC) is a nanostructured material with unique properties and wide applicability. In order to decrease the production cost of bacterial cellulose, lignocellulose-based media have considerable potential as alternative cost-effective feedstocks. However, pretreatment and enzymatic hydrolysis of lignocellulose to sugars also generate fermentation inhibitors. Detoxification of lignocellulosic hydrolysates is needed to achieve efficient production of BC. In this investigation, different methods for detoxification of spruce hydrolysate prior to production of BC were compared with respect to effects on potential inhibitors and fermentable sugars, sugar consumption, BC yield, and cell viability. The objectives were to identify efficient detoxification methods and to achieve a better understanding of the role played by different inhibitors in lignocellulosic hydrolysates.

**Results:**

In a first series of experiments, the detoxification methods investigated included treatments with activated charcoal, alkali [sodium hydroxide, calcium hydroxide (overliming), and ammonium hydroxide], anion and cation ion-exchange resins, and reducing agents (sodium sulfite and sodium dithionite). A second series of detoxification experiments included enzymatic treatments (laccase and peroxidase). The potential inhibitors studied included aliphatic acids, furan aldehydes, and phenolic compounds. The best effects in the first series of detoxification experiments were achieved with activated charcoal and anion exchanger. After detoxification with activated charcoal the BC yield was 8.2 g/L, while it was 7.5 g/L in a reference medium without inhibitors. Treatments with anion exchanger at pH 10 and pH 5.5 gave a BC yield of 7.9 g/L and 6.3 g/L, respectively. The first series of experiments suggested that there was a relationship between the BC yield and phenolic inhibitors. Therefore, the second series of detoxification experiments focused on treatments with phenol-oxidizing enzymes. The BC yield in the laccase-detoxified hydrolysate reached 5.0-5.5 g/L after 14 days cultivation, which demonstrated the important inhibitory role played by phenolic compounds.

**Conclusions:**

The investigation shows that detoxification methods that efficiently remove phenolics benefit bacterial growth and BC production. Negative effects of salts could not be excluded and the osmotolerance of *Gluconacetobacter xylinus* needs to be further investigated in the future. Combinations of detoxification methods that efficiently decrease the concentration of inhibitors remain as an interesting option.

## Background

Bacterial cellulose (BC) is an extracellular biopolymer product of acetic acid bacteria, principally of the genus *Gluconacetobacter* (formerly *Acetobacter*). More recently *Gluconacetobacter xylinus* has attracted special attention due to its potential for commercial production of BC. Compared to plant cellulose, the nanofibril network of BC has interesting properties such as excellent water-holding capacity, high degree of polymerization, high crystallinity, high purity, good biocompatibility, and excellent mechanical properties [1]. Therefore, BC has a wide application area that includes biomedical materials, health foods, high-quality audio membranes, functional paper, fuel cells, and textiles [1-4]. Static bacterial cultures are preferred for commercial production of BC, but have some shortcomings including low efficiency and high production cost. In order to decrease the production costs of BC, cultivation on media based on relatively inexpensive feedstocks has been investigated. These feedstocks include agricultural residues and waste materials, such as wheat straw [5,6], konjak glucomannan [7], cotton-based waste textiles [8], and waste fiber sludge [9]. The feedstock is hydrolyzed by using acid or by using pretreatment and enzymatic hydrolysis. Acid hydrolysis is inexpensive and efficient but the sugar yields may be relatively low due to incomplete hydrolysis of cellulose or due to by-product formation. Enzymatic hydrolysis is generally considered advantageous since high sugar yields can be achieved [9]. Residues from forestry (branches, tops, sawdust, etc.) offer an alternative to agricultural feedstocks and waste. Softwood (conifer) forests are a major source of lignocellulosic feedstock in the northern hemisphere. For example, 82% of the forest resources of Sweden consist of softwood, mainly Norway spruce and Scots pine [10]. Softwood residues, which include spruce wood, are rich in carbohydrates such as glucan, mannan, and galactan, which is advantageous considering that these polysaccharides can be hydrolyzed to hexose sugars, which are typically well suited as substrates in microbial fermentation processes.

It is well-known that lignocellulosic hydrolysates contain fermentation inhibitors that are formed during pretreatment at high temperature and low pH. These inhibitors include furan aldehydes [furfural and 5-hydroxymethyl-furfural (HMF)], aliphatic acids (such as acetic acid, formic acid, and levulinic acid), and phenolic compounds [11]. During production of BC using media based on hydrolysates from wheat straw and konjak glucomannan fermentation inhibition was experienced and detoxification had to be employed [5,7].

Various chemical, physical, and biological methods have been employed for detoxification of lignocellulosic hydrolysates. These methods include treatments with alkali (NaOH, Ca(OH)_2_ and NH_4_OH), ion-exchange resins, reducing agents (such as sodium sulfite and sodium dithionite), activated charcoal, and enzyme (laccase and peroxidase) [11]. It is important to identify detoxification processes that are efficient and inexpensive, and which are also compatible with other process steps.

In this study, an enzymatic hydrolysate of SO_2_-pretreated spruce wood was evaluated with regard to fermentability for BC production with the bacterium *G. xylinus*. The effects of different detoxification methods were compared by analyzing the impact on the concentrations of potential fermentation inhibitors and sugars, the yield of BC, sugar consumption, and cell viability. The aims were to identify efficient detoxification methods and lignocellulose degradation products that inhibit *G. xylinus*.

## Results and discussion

### Detoxification of spruce hydrolysate

The three times diluted spruce hydrolysate was referred to as untreated hydrolysate and contained 19.87 g/L glucose, 4.35 g/L mannose, 3.77 g/L xylose, 0.97 g/L galactose, and 0.69 g/L arabinose. The concentrations of HMF and furfural were roughly equal and amounted to 0.5 g/L of each. The concentration of acetic acid was 1.72 g/L, and there was also a small amount of formic acid (0.18 g/L). The total concentration of phenolic compounds was estimated to 1.3 g/L.

**Figure 1:**
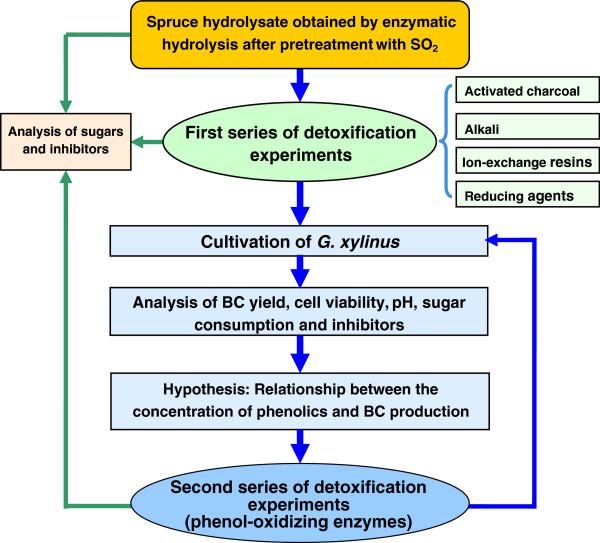
Flowchart of experimental work.

In a first series of experiments (Figure 1), ten different detoxification treatments were performed as summarized in Table 1. The results of the chemical analysis of sugars and fermentation inhibitors after the treatments are also shown in Table 1. The results show that activated charcoal treatment did not affect the sugar concentration very much, but instead removed most of the furan aldehydes and the phenols. The acids also decreased, but about two thirds remained (Table 1). Among the different treatments, the activated charcoal treatment was most efficient in removing furans (94% of 1.0 g/L), acetic acid (28% of 1.72 g/L), formic acid (39% of 0.18 g/L), and total phenolics (88% of 1.3 g/L). The removal efficiency was slightly higher than achieved in previous investigations [12], where treatment of a brewery’s spent grain hydrolysate with activated charcoal resulted in removal of 92% of the furfural, 68% of the HMF, 17% of the acetic acid, 11% of the formic acid, and 58% of the phenolics. In our study, the removal of furan aldehydes decreased in the order: activated charcoal (94%) > overliming (35-45%) > anion exchanger at pH 10 (22-26%) > cation exchanger at pH 10 (12-15%) > NH_4_OH (10-15%) > anion exchanger at pH 5.5 (6-9%), NaOH (8%) > cation exchanger at pH 5.5 (4-6%). For aliphatic acids, the efficiency decreased in the order: activated charcoal (28-39%) > anion exchanger at pH 10 (22-33%) > anion exchanger at pH 5.5 (20-28%) > cation exchanger at pH 10 (10%) > cation exchanger at pH 5.5 (7-9%) > NaOH (2-6%). As expected, the treatments with Ca(OH)_2_ and NH_4_OH did not lead to removal of aliphatic acids. For total phenols, the removal efficiency decreased in the order: activated charcoal (88%) > anion exchanger at pH 10 (79%) > anion exchanger at pH 5.5 (53%) > cation exchanger at pH 10 (22%) > Ca(OH)_2_ (14%) > cation exchanger at pH 5.5 (8%), NH_4_OH (8%) > NaOH (1%). The reducing agents (sodium sulfite and \sodium dithionite) apparently did not decrease the concentrations of any of the inhibitors or group of inhibitors measured, which agrees with previous results [13]. However, Cavka et al. [14] have shown that sulfite and dithionite can sulfonate both phenolics and furan aldehydes in model reactions. If the furan aldehydes had been affected by the treatment with the reducing agents, this would have been detected in the analysis since sulfonation renders furan aldehydes highly hydrophilic and changes the elution time on a C18 column. As the phenols were analyzed with group analysis based on a spectrophotometric assay, it is not certain that sulfonation of phenolics would be detected, since sulfonation primarily targets other groups than phenolic hydroxyl groups. Therefore, even if the concentration of phenolics was not affected by treatment with reducing agents (Table 1), the toxicity of the phenolics may nevertheless have been changed by the treatment.

**Table 1:** Analyses of sugars and inhibitors in the spruce hydrolysate after detoxification

**No.**	**Detoxification**	**Sugars and inhibitors (% of concentration in untreated samples) **^**a**^									
		**Glucose**	**Mannose**	**Xylose**	**Galactose**	**Arabinose**	**HMF**	**Furfural**	**Acetic acid**	**Formic acid**	**Total phenols**
1	Activated charcoal	96	99	95	98	88	**6**	**6**	**72**	**61**	**12**
2	NaOH	97	99	94	100	90	92	92	98	94	99
3	Ca(OH)_2_	87	97	90	94	84	**55**	**65**	100	111	86
4	NH_4_OH	88	98	93	97	83	85	90	100	102	92
5	Anion exchanger, pH 10	88	90	88	84	77	**74**	**78**	**78**	**67**	**21**
6	Anion exchanger, pH 5.5	90	93	98	95	94	91	94	**80**	**72**	**47**
7	Cation exchanger, pH 10	91	88	87	97	91	85	88	90	90	78
8	Cation exchanger, pH 5.5	93	95	102	101	98	94	96	93	91	92
9	Sodium sulfite	100	98	94	102	99	100	102	99	106	100
10	Sodium dithionite	98	99	100	103	103	101	104	102	106	100
11	Untreated ^a^	100	100	100	100	100	100	100	100	100	100

^a^ The concentration of each sugar and inhibitor in the spruce hydrolysate without detoxification was set to 100%, which corresponds to 19.87 g/L glucose, 4.35 g/L mannose, 3.77 g/L xylose, 0.97 g/L galactose, 0.69 g/L arabinose, 0.53 g/L HMF, 0.51 g/L furfural, 1.72 g/L acetic acid, 0.18 g/L formic acid, and 1.30 g/L phenolic compounds.

Treatment with activated charcoal was found to be one of the most promising methods for detoxification of wood hydrolysates prior to fermentation with the yeast *Debaryomyces hansenii* although the chemical effect of the treatment was not further investigated [15]. Detoxification with anion-exchange resins at pH 10 has been reported as a very efficient method to decrease the concentrations of furan derivatives and total phenols in spruce hydrolysates [16,17]. Obviously, it was also efficient in removing aliphatic acids, ending up as the second most efficient method after activated charcoal (Table 1). Compared to anion exchanger, cation exchanger treatments were much less efficient in removing fermentation inhibitors, which agrees well with previous results [17,18]. Overliming of hydrolysate led to 35-45% removal of furans and 14% removal of phenolics, but did not cause any change of the levels of aliphatic acids. Among the three alkaline treatments, overliming had the largest effect. After detoxification with alkali, the concentrations of aliphatic acids (acetic and formic acid) either increased or remained very close to the initial levels, which is similar to the results reported in previous studies [19,20]. The increase of aliphatic acids can be attributed to the importance of sugars as precursors to aliphatic acids in alkaline degradation of sugar [21]. As shown in Table 1, the largest decrease in the concentrations of monosaccharides (mainly affecting glucose and arabinose) was observed after treatment with overliming (13% for glucose and 16% for arabinose), NH_4_OH (12% for glucose and 17% for arabinose), anion exchanger at pH 10 (12% for glucose and 23% for arabinose) and cation exchanger at pH 10 (around 10% for both glucose and arabinose). The effects of other detoxification methods on the concentration of monosaccharides and inhibitors were negligible, especially with regard to the treatments with dithionite and sulfite. Alriksson et al. [13] also found that the addition of dithionite or sulfite did not affect the concentrations of monosaccharides in hydrolysates of spruce wood and sugarcane bagasse. Nevertheless, addition of 5 mM dithionite or 10 mM sulfite resulted in major improvements of the fermentability of hydrolysates using *Saccharomyces cerevisiae* as the fermenting microorganism. The reason behind the improvement of fermentability, though the total phenolic content remains the same before and after treatment, is assumed to be that the treatment converts phenolics and other aromatics to less toxic compounds by making reactive compounds inert and by introducing strongly hydrophilic sulfonate groups [14].

### BC production by using hydrolysates

Spruce hydrolysates prepared by using 10 different detoxification methods were used for the production of bacterial cellulose by *Gluconacetobacter xylinus* ATCC 23770. For the purpose of evaluating the effectiveness of the detoxification, cultivations using untreated hydrolysate and a reference medium containing the same concentrations of monosaccharide sugars as the untreated hydrolysate, but no inhibitors, were also performed.

**Figure 2:**
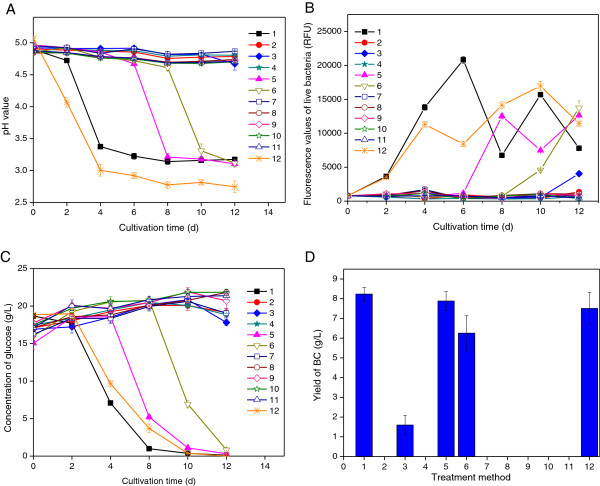
**Effects of different detoxification methods on cultures of *G. Xylinus*.** The figure shows the time course of the pH value **(A)**, the fluorescence values of live bacteria **(B)**, the residual glucose **(C)**, and the yield of BC after 12 days of cultivation **(D)**. Treatment methods 1–10 represent culture media based on hydrolysate detoxified with 10 different methods and correspond to the detoxification methods numbered in Table 1; method 11 represents untreated hydrolysate, and method 12 represents a reference medium without inhibitors. Mean values and standard deviations from three separate experiments are given.

Figure 2 shows differences for controls and cultures with detoxified medium with regard to the pH value of the culture medium (A), the cell viability (B), the concentration of residual glucose (C), and the yield of BC (D). For hydrolysates treated with activated charcoal (No. 1), anion exchanger at pH 10 (No. 5), anion exchanger at pH 5.5 (No. 6), and overliming (No. 3), there were clear differences compared to cultures with untreated hydrolysate. The cultivations in hydrolysate medium can be compared to the cultivation in reference medium without inhibitors (No. 12). Figure 2D shows that BC production was achieved only in the four hydrolysates mentioned above (No. 1, 3, 5 and 6) and in the reference medium. The BC yield decreased in the following order: activated charcoal (8.2 g/L) > anion exchanger at pH 10 (7.9 g/L) > reference medium (7.5 g/L) > anion exchanger at pH 5.5 (6.3 g/L) > overliming (1.6 g/L). The changes in the proliferation of the bacterial cells (Figure 2B) and in the consumption of glucose (Figure 2C) correspond very well with the BC yield. The fluorescence value of live bacteria from activated charcoal-treated hydrolysate was higher than for the reference medium during the first six days of the cultivation (Figure 2B). This might be due to that the level of carbon source was higher in the activated charcoal-treated hydrolysate since the reference medium only contained the five main sugars. Components and effects that could tentatively contribute include oligosaccharides from enzymatic hydrolysis, acetic acid released from hemicellulose, and synergistic effects of sugars affecting the metabolism [6]. The culture based on overliming-treated hydrolysate started to change at the very end of the experiment. The results clearly indicated that the hydrolysate detoxified with activated charcoal treatment was best suited for growth of *G. xylinus* and production of BC. The second most efficient detoxification was the treatment with anion exchanger at pH 10 and 5.5, both of which resulted in considerably better fermentability than treatment with overliming. Hong et al. [5,7] used treatments with alkali (NaOH, Ca(OH)_2_ and NH_4_OH) combined with activated charcoal for detoxification of acid hydrolysates of konjak glucomannan and wheat straw and showed that a combination of overliming and treatment with activated charcoal gave the best results. However, these studies did not clarify which individual detoxification method that was the most efficient one or which chemical effects the different detoxification methods have. The current work shows that activated charcoal is the best treatment for BC production and explains the causes that lie behind it.

The results regarding the detoxification efficiency obtained for BC production in the spruce hydrolysates seem to partially differ from those reported for ethanolic fermentation with yeast. Alkali treatments, including overliming, are usually among the best methods for ethanol production with *S. cerevisiae* and *Candida shehatae*[16,22,23], but were not that successful for BC production (this work). Addition of dithionite or sulfite to lignocellulosic hydrolysates efficiently detoxified hydrolysates for ethanol production with *S. cerevisiae*[13,14], but did not improve BC production with *G. xylinus*. Treatment with activated charcoal was found to work well both for the yeasts *Debaryomyces hansenii*[15] and *C. shehatae*[23], but also for *G. xylinus* (this work). Together with the need to dilute the hydrolysate for cultivation of *G. xylinus*, all these observations suggest that yeasts such as *S. cerevisiae* are more tolerant to inhibition and that *G. xylinus* is more sensitive to addition of salts (such as alkali and sodium salts of sulfur oxyanions) than *S. cerevisiae.* It is a possibility that an important aspect of dilution is that it reduces the ionic strength, which may be more important for *G. xylinus* than for *S. cerevisiae*. It has been shown that *G. xylinus* can tolerate an addition of at least 100 mM sodium sulfate, but not more than 20 mM sodium chloride [5]. *Gluconacetobacter diazotrophicus* PAl 5 was reported to be resistant to high concentrations of cadmium chloride, cobalt chloride and zinc chloride with minimum inhibitory concentrations of 1.2, 20 and 20 mM, respectively [24]. In comparison, *Escherichia coli* can tolerate 2.5-3% (w/v) sodium chloride (roughly 400–500 mM) in cheese [25]. Nagata et al. [26] reported that the respiratory activity of *E. coli* K-12 and ATCC 9637 decreased with increasing sodium chloride concentration. When the concentration of the sodium chloride solution was 1 M, no living *E. coli* cells were found in the culture media after 24 h cultivation [26]. *S. cerevisiae* is known to be relatively osmotolerant and can be grown in a 1.5 M solution of sodium chloride [27]. Thus, compared to other microorganisms, such as *E. coli* and *S. cerevisiae*, *G. xylinus* has relatively poor capacity to tolerate sodium chloride. In order to adjust the pH to 5.0, a solution of sodium hydroxide was added to the spruce hydrolysate and this would result in about 125 mM of sodium chloride. Therefore, benefits achieved by conversion of inhibitors, such as phenols and furans, may be lost by salt addition for alkali treatments and treatments with sulfur oxyanions. The result that calcium hydroxide treatment gave positive results while treatments with sodium hydroxide and ammonium hydroxide did not (Figure 2) also points in this direction, since calcium tends to form sparsely soluble salts, for example with ions such as sulfate and phosphate, while sodium and ammonium do not. If salt precipitation occurs, the ionic strength would be lower than otherwise, as shown in experiments with ethanologenic *E. coli*[28]. The osmotolerance of *G. xylinus* requires further investigation in the future.

In order to evaluate the consumption of sugars and changes in the concentrations of fermentation inhibitors during cultivation, the concentrations of five monosaccharides and four inhibitors were measured before cultivation (after autoclaving) and after the cultivation was finished. The results for the sugars are shown in Table 2. The results show that glucose was the main nutrient source and that it was consumed efficiently in all cultivations. The result also suggests that some of the xylose and mannose were consumed, but that the consumption of arabinose and galactose was negligible. Dahman et al. [29] suggested that *G. xylinus* has the ability to utilize xylose and mannose, and the yield of BC from mannose-based culture medium was higher than that from xylose-based culture medium. There are some reports that certain *G. xylinus* strains have poor capability to utilize arabinose and galactose. Mikkelsen et al. [30] reported that galactose appeared to be the least suitable carbon source for bacterial cellulose production. Keshk and Sameshima [31] found that the cellulose yield in arabinose- or galactose-based media was only around 22% of that in glucose-based medium.

**Table 2:** Monosaccharide concentrations in the spruce hydrolysate before and after cultivation

**A. Prior to cultivation of *G. xylinus***						
**No.**	**Treatment**	**Glucose (g/L)**	**Mannose (g/L)**	**Xylose (g/L)**	**Galactose (g/L)**	**Arabinose (g/L)**
1	Activated charcoal	18.7 ± 0.2	4.1 ± 0.3	3.3 ± 0.4	0.9 ± 0.1	0.6 ± 0.1
2	NaOH	17.1 ± 0.4	4.1 ± 0.3	3.3 ± 0.3	0.8 ± 0.1	0.6 ± 0.1
3	Ca(OH)_2_	16.9 ± 0.8	4.0 ± 0.3	3.2 ± 0.2	0.9 ± 0.1	0.6 ± 0.1
4	NH_4_OH	17.5 ± 0.7	4.0 ± 0.2	3.3 ± 0.3	0.9 ± 0.1	0.6 ± 0.1
5	Anion exchanger, pH 10	15.1 ± 0.8	3.2 ± 0.6	2.6 ± 0.6	0.7 ± 0.1	0.4 ± 0.1
6	Anion exchanger, pH 5.5	16.0 ± 0.9	3.6 ± 0.5	3.0 ± 0.5	0.7 ± 0.1	0.5 ± 0.1
7	Cation exchanger, pH 10	16.2 ± 0.9	3.8 ± 0.3	3.1 ± 0.3	0.8 ± 0.1	0.5 ± 0.1
8	Cation exchanger, pH 5.5	16.8 ± 0.7	3.9 ± 0.4	3.3 ± 0.5	0.9 ± 0.1	0.5 ± 0.1
9	Sodium sulfite	17.7 ± 0.2	3.7 ± 0.5	3.2 ± 0.4	0.8 ± 0.1	0.5 ± 0.1
10	Sodium dithionite	17.5 ± 0.1	3.9 ± 0.4	3.3 ± 0.5	0.8 ± 0.1	0.6 ± 0.1
11	Untreated	17.1 ±0.3	3.9 ± 0.2	3.3 ± 0.2	0.8 ± 0.1	0.6 ± 0.2
12	Reference	18.8 ± 0.1	4.1 ± 0.1	3.1 ± 0.1	0.8 ± 0.1	0.6 ± 0.1
**B. After cultivation of *G. xylinus***						
**No.**	**Treatment**	**Glucose (g/L)**	**Mannose (g/L)**	**Xylose (g/L)**	**Galactose (g/L)**	**Arabinose (g/L)**
1	Activated charcoal	**0.1 ± 0.1**	2.5 ± 0.3	2.2 ± 0.1	0.7 ± 0.1	0.5 ± 0.1
2	NaOH	19.1 ± 0.6	3.6 ± 0.3	3.1 ± 0.3	0.8 ± 0.1	0.6 ± 0.1
3	Ca(OH)_2_	17.8 ± 0.4	3.9 ± 0.2	3.3 ± 0.3	0.8 ± 0.1	0.5 ± 0.1
4	NH_4_OH	18.9 ± 0.2	3.6 ± 0.1	3.1 ± 0.1	0.8 ± 0.1	0.5 ± 0.1
5	Anion exchanger, pH 10	**0.3 ± 0.1**	2.3 ± 0.1	2.1 ± 0.1	0.7 ± 0.1	0.4 ± 0.1
6	Anion exchanger, pH 5.5	**0.8 ± 0.3**	2.5 ± 0.1	2.4 ± 0.1	0.7 ± 0.1	0.4 ± 0.1
7	Cation exchanger, pH 10	19.0 ± 0.6	2.4 ± 0.3	2.3 ± 0.4	0.7 ± 0.1	0.5 ± 0.1
8	Cation exchanger, pH 5.5	21.8 ± 0.3	3.3 ± 0.1	3.2 ± 0.1	0.8 ± 0.1	0.6 ± 0.1
9	Sodium sulfite	20.7 ± 1.2	3.6 ± 0.1	3.1 ± 0.4	0.8 ± 0.1	0.5 ± 0.1
10	Sodium dithionite	21.8 ± 0.4	3.9 ± 0.1	3.7 ± 0.1	0.9 ± 0.1	0.5 ± 0.1
11	Untreated	21.4 ± 0.6	4.0 ± 0.3	3.3 ± 0.2	0.8 ± 0.2	0.6 ± 0.1
12	Reference	**0.1 ± 0.1**	2.6 ± 0.1	1.9 ± 0.1	0.8 ± 0.1	0.5 ± 0.1

For those cultures that did not produce BC, the concentration of sugars did not decrease but instead increased slightly after cultivations. This can be attributed to the water evaporation of culture media during cultivation over an extended period of time.

**Figure 3:**
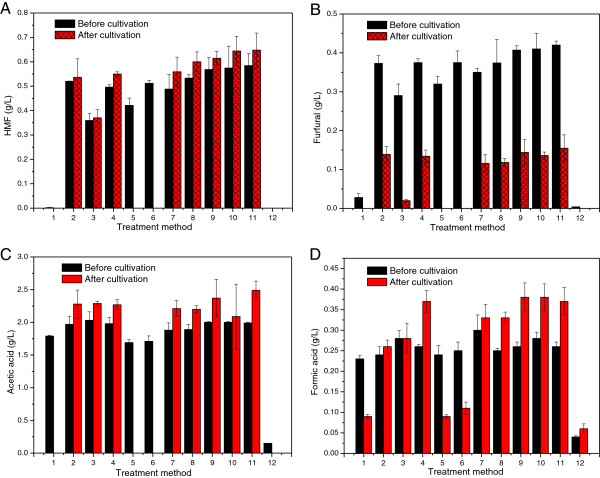
**Concentrations of HMF (A), furfural (B), acetic acid (C) and formic acid (D) before and after cultivation.** Methods 1–11 represent cultures that correspond to the treatments numbered in Table 2. Method 12 represents reference medium without inhibitors. Mean values and standard deviations from three separate experiments are given.

Since medium components (such as amino acids, peptides and protein) in the culture media would interfere with the assay of total phenols using the Folin–Ciocalteu reagent, only the concentrations of furan aldehydes and aliphatic acids are included in Figure 3. As shown in Figure 3A and 3B, in 12 days all residual HMF and furfural had disappeared from the hydrolysate detoxified with activated charcoal, anion exchanger at pH 10, and anion exchanger at pH 5.5. In these cases, *G. xylinus* grew well in the culture medium (Figure 2B). Therefore it is probable that *G. xylinus* is able to convert furfural, perhaps to furoic acid or to furfuryl alcohol, during the cultivation. Under aerobic conditions, furfural can to some extent be oxidized to furoic acid by the yeast *S. cerevisiae*[32]. Furfural can also be reduced by yeast to furfuryl alcohol [32]. Some methanobacteria have the capacity to reduce furfural to furfuryl alcohol [33]. It has been reported that HMF is also reduced by *S. cerevisiae* and that the main conversion product is 5-hydroxymethylfurfuryl alcohol [34]. The possibility that *G. xylinus* also can convert furfural and HMF needs to be investigated further. With regard to cultures in which there was no BC production, the concentrations of HMF, acetic acid and formic acid increased slightly after cultivation, as previously noticed for the sugars (Table 2). This can be attributed to evaporation of water during cultivations performed under extended periods of time. However, more than half of the concentration of furfural was lost even for cultures in which there was no BC production (Figure 3B). The explanation is that furfural evaporates more easily than HMF. Furfural has been reported to be partially (37%) removed by evaporation of 10% of the initial volume of an acid hydrolysate of spruce and was completely removed by evaporation of 90% of the initial volume [16]. For HMF, evaporation of 90% of the initial volume led only to a decrease by 4% [16], which shows that it is much less volatile. The decrease in the concentration of furfural in cultures that produced BC is probably due to a combination of bioconversion and evaporation. All residual acetic acid was exhausted after cultivation of hydrolysates detoxified with activated charcoal, anion exchanger at pH 10, and anion exchanger at pH 5.5 (Figure 3C). More than half of the formic acid in these three hydrolysates was consumed (Figure 3D). It has been reported that acetic acid can be used as an energy source that highly improves the cellulose yield [35]. It is expected that acetic acid would be more easily metabolized than formic acid by *G. xylinus*. For the reference medium, small amounts of furfural, acetic acid and formic acid were produced during autoclaving, which can be attributed to sugar degradation during thermal treatment.

### Inhibitory effect of phenolic compounds

**Figure 4:**
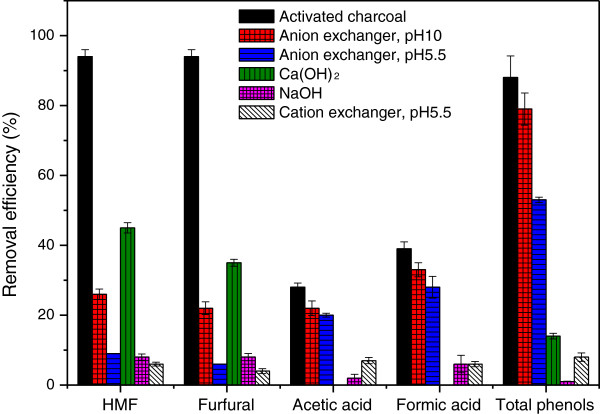
**Removal efficiency of five potential inhibitors in the spruce hydrolysate.** Mean values and standard deviations from three separate experiments are given.

According to Figure 2D, the BC yields obtained in the hydrolysates decreased in the following order: activated charcoal > anion exchanger at pH 10 > anion exchanger at pH 5.5 > overliming. This order agrees with the order of removal of total phenols and aliphatic acids, but not of furan aldehydes (Table 1 and Figure 4). Although overliming resulted in the second best removal of furan aldehydes (Figure 4; removal of 45% HMF and of 35% furfural), bacterial cells started to grow very late (Figure 2B) and the BC yield was only 1.6 g/L, much lower than for the other three detoxified hydrolysates (Figure 2D). The results suggest that furan aldehydes might not be the key fermentation inhibitors for *G. xylinus*. In contrast, there was a large difference in total phenolic compounds after detoxification for the hydrolysates used in cultures that did result in high yields of BC. As shown in Figure 2, the hydrolysate treated with activated charcoal started to produce BC on the second day of fermentation. This was also the most effective method to reduce the total phenolic compounds (88%). Treatments with anion exchanger at pH 10 and pH 5.5 caused reduction of 79% and 53% of the total phenols, respectively. This suggested that there is a relationship between the concentration of phenolics and BC production. In order to investigate this hypothesis, a second experimental series was performed.

Previous studies show that treatments with phenol-oxidizing enzymes specifically remove phenolic compounds. Specifically, treatment with laccase was the only detoxification method that removed only one group of inhibitors, the phenolic compounds [16,23,36]. The effect of the enzymatic treatment on the phenols has also been elucidated [36]. Therefore, in a second series of experiments treatments with laccase and horseradish peroxidase were carried out to investigate the role of the phenolics (Figure 1). Table 3 shows the effect of enzymatic detoxification on the composition of the spruce hydrolysate. Compared to the controls, laccase and peroxidase treatment removed total phenolics by 46-55% and 25%, respectively. The treatments did not significantly affect the contents of furan aldehydes, aliphatic acids, and sugars (Table 3). The decrease in the contents of total phenolics was almost the same in the hydrolysate treated with 11.8 and 59 mg peroxidase. This might be due to the limited dosage of hydrogen peroxide.

**Table 3:** Influence of enzymatic detoxification on the composition of the spruce hydrolysate

**A. Laccase treatment (g/L)**										
**Enzymatic treatment**	**Glucose**	**Xylose**	**Mannose**	**Arabinose**	**Galactose**	**HMF**	**Furfural**	**Acetic acid**	**Formic acid**	**Total phenols**
Lac-88 ^a^	19.7 ± 0.5	3.4 ± 0.1	3.7 ± 0.1	0.5 ± 0.1	0.5 ± 0.1	0.5 ± 0.1	0.5 ± 0.1	1.7 ± 0.2	0.2 ± 0.1	**0.6 ± 0.1**
Lac-440 ^a^	20.8 ± 0.2	3.5 ± 0.1	4.0 ± 0.1	0.4 ± 0.1	0.5 ± 0.1	0.5 ± 0.1	0.5 ± 0.1	1.6 ± 0.1	0.2 ± 0.1	**0.5 ± 0.1**
Lac-control ^b^	18.7 ± 0.9	3.1 ± 0.1	3.9 ± 0.2	0.4 ± 0.1	0.6 ± 0.1	0.5 ± 0.1	0.5 ± 0.1	1.7 ± 0.1	0.2 ± 0.1	1.1 ± 0.1
**B. HRP treatment (g/L)**										
**Enzymatic treatment**	**Glucose**	**Xylose**	**Mannose**	**Arabinose**	**Galactose**	**HMF**	**Furfural**	**Acetic acid**	**Formic acid**	**Total phenols**
HRP-11.8 ^c^	19.1 ± 0.4	3.2 ± 0.1	3.7 ± 0.1	0.4 ± 0.1	0.5 ± 0.1	0.5 ± 0.1	0.5 ± 0.1	1.7 ± 0.1	0.2 ± 0.1	**0.9 ± 0.1**
HRP-59 ^c^	19.7 ± 0.5	3.3 ± 0.1	4.1 ± 0.1	0.4 ± 0.1	0.6 ± 0.1	0.5 ± 0.1	0.5 ± 0.1	1.6 ± 0.1	0.2 ± 0.1	**0.9 ± 0.1**
HRP-control ^b^	18.4 ± 0.7	3.6 ± 0.3	3.7 ± 0.3	0.4 ± 0.1	0.5 ± 0.1	0.5 ± 0.1	0.5 ± 0.1	1.7 ± 0.1	0.2 ± 0.1	1.2 ± 0.1

^a^ Lac-88 and Lac-440 represent the hydrolysates that were treated with 88 mg and 440 mg laccase, respectively.

^b^ Lac-control and HRP-control represent the control hydrolysates for the treatments with laccase and horseradish peroxidase, respectively.

^c^ HRP-11.8 and HRP-59 represent the hydrolysates that were treated with 11.8 mg and 59 mg horseradish peroxidase, respectively.

**Figure 5:**
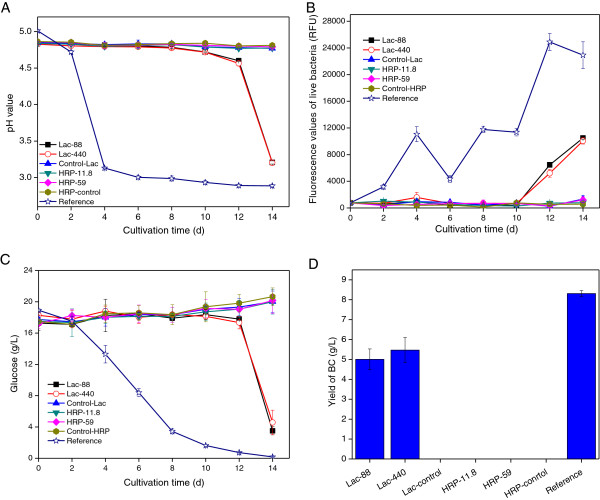
**Effects of enzymatic treatments on cultures of *G. Xylinus*.** The figure shows the time course of the pH value **(A)**, the fluorescence values of live bacteria **(B)**, the residual glucose **(C)**, and the yield of BC after 14 days of cultivation **(D)**. Mean values and standard deviations from three separate experiments are given.

In order to evaluate the fermentability of the enzyme-treated hydrolysates, the hydrolysates listed in Table 3 and a reference medium were used for BC production and the results are shown in Figure 5. Changes in pH value, contents of live bacterial cells, and consumption of glucose showed a similar tendency as observed in previous experiments (Figure 5A, B and C). In the beginning of the cultivations, changes only occurred in the reference medium, which had the same amount of sugars as the untreated hydrolysate. After about 10 days, the cultures based on laccase-treated hydrolysates started to grow (Figure 5B) and consume glucose (Figure 5C). However, the cultures based on other hydrolysates did not show any changes during 14 days of cultivation. Figure 5D shows that BC was only produced in the laccase-treated hydrolysates and in the reference medium. The yields of BC in the hydrolysates treated with 88 and 440 mg of enzyme preparation were 5.0 g/L and 5.5 g/L, respectively. In comparison, the culture based on reference medium gave 8.3 g/L.

Experiments with laccase-treated hydrolysates showed that BC could be produced when a certain fraction of the phenolic compounds (0.6 g/L, Table 3) was removed from the spruce hydrolysate. For better detoxification effects and significant increased fermentability of hydrolysate for BC production, it is possible that a combination of laccase treatment and overliming could be applied. Hong et al. [5,7] reported that BC can be obtained from the laccase-treated acid hydrolysates of konjac glucomannan and wheat straw. This indicates that phenolic compounds were very important fermentation inhibitors in the production of BC by *G. xylinus*, while furan aldehydes and aliphatic acids probably play a less important role.

The results for *G. xylinus* growth and BC production obtained with the spruce hydrolysates differed from previous detoxification studies on ethanologenic microorganisms. It is reasonable that the tolerance towards inhibitors is different for microorganisms that are adapted to different environments. Some researchers maintain that even small concentrations of HMF and furfural have a detrimental effect on ethanolic fermentation processes [37,38]. Klinke et al. [39] found that the ethanol production of *Zymomonas mobilis* CP4 (pZB5) was generally more inhibited than it was for other microbes that were tested in cultures with relatively low concentrations of acids and furan aldehydes. *E. coli* was found to be very tolerant to furfural and HMF compared to *Pichia stipitis*, *Z. mobilis* and *Candida shehatae*[39]. In our study, BC could be produced from culture medium containing 0.5 g/L HMF and 0.5 g/L furfural, also in the presence of other fermentation inhibitors (Table 3 and Figure 5D). Phenolic compounds have been found to have a considerable inhibitory effect in the fermentation of lignocellulosic hydrolysates, and low molecular weight phenolic compounds are considered to be most toxic. It is assumed that phenolic compounds affect ethanol fermentation by disturbing the capability of the biological membrane to serve as a selective barrier, which would cause reduction of both cell growth and ethanol production [38]. While the treatments with laccase removed about 50% of the phenols, the treatments with peroxidase removed only about 25%, which was not sufficient to detoxify the hydrolysate. The difference may be due either to that it was important to decrease the total load of toxic phenols below a certain threshold to permit growth or to that the phenols that are most easily oxidized are not the most toxic ones. Previous studies show that some phenols are oxidized more rapidly than others during enzymatic treatment [36] and also that different phenolics have very different toxic effect on the fermenting microorganism [40]. The effect of phenolic compounds on cell growth and BC production of *G. xylinus* is not well understood and the mechanism of inhibition by phenolic compounds requires further investigation.

## Conclusions

Treatment with activated charcoal resulted in the most efficient detoxification and gave the best fermentability, and was followed in order of efficiency by the treatments with anion exchanger, laccase, and Ca(OH)_2_. Results with the phenol-oxidizing enzyme laccase showed that good fermentability could be achieved if a certain fraction of phenolic compounds was removed from the lignocellulosic hydrolysate. The effect of specific inhibitory compounds on *G. xylinus*, especially phenolic compounds, warrants attention in future studies. The fact that the yields of BC from hydrolysates treated with activated charcoal and anion exchanger at pH 10 were higher than the BC yield of cultures based on a reference medium containing the same amount of the five predominant sugars as the untreated hydrolysate suggests the presence of hydrolysate components that stimulate BC production, a phenomenon that needs to be elucidated in further research efforts. Strong inhibition or no growth was found when the spruce hydrolysate was treated with NaOH, NH_4_OH or reducing sulfur oxyanions. Negative effects of salts could not be excluded in this context and the osmotolerance of *G. xylinus* needs to be further investigated in the future.

The choice of detoxification method would depend on the composition of the hydrolysates as well as on the fermenting microorganism. For the production of BC with *G. xylinus* in spruce hydrolysate, the detoxification methods that efficiently remove phenolics benefit bacterial growth and BC production. Combinations of methods that efficiently remove inhibitors, for example treatments with activated charcoal and anion exchanger, remain as an interesting option.

## Methods

### Preparation of hydrolysate

An overview of the investigation is presented in Figure 1. Spruce hydrolysate was produced from pretreated wood chips of Norwegian spruce (*Picea abies*). The pretreatment was performed in the Swedish biorefinery demonstration plant Etanolpiloten (SEKAB, Örnsköldsvik, Sweden). Unbarked wood chips (moisture content ~50%) were treated in a continuous mode with sulfur dioxide in a half-filled 30-litre reactor and at a pressure of 18 bar (204°C). The wood chips were impregnated with 1.2-1.3 kg SO_2_/h, which corresponds to 1% SO_2_/kg of spruce wood chips [DW (dry-weight)]. The residence time was 7–8 min, and the resulting pH of the slurry was 1.4-1.5.

Prior to hydrolysis, the pH of the spruce slurry was adjusted to 5.1 with a 5 M solution of sodium hydroxide. Each of six 2-L shake flasks was filled with 950 g of spruce slurry. The suspended solids (SS) content of the spruce slurry was 14.1%. Commercially available preparations of cellulase and cellobiase were added to the slurries. The cellulase preparation, which was from *Trichoderma reesei* ATCC 26921, had a stated activity of 700 endoglucanase units (EGU)/g (Sigma-Aldrich, Steinheim, Germany) and the loading was 319 EGU/g of solids (DW). The cellobiase preparation, Novozyme 188, had a stated activity of 250 cellobiase units (CBU)/g (Sigma-Aldrich) and the loading was 23 CBU/g of solids (DW). After addition of enzymes, the slurries were incubated with shaking (Kuhner Lab-Therm LT-X, A. Kühner AG, Birsfelden, Switzerland) at 45°C and 110 rpm for 72 h. After hydrolysis, the slurries were centrifuged (Allegra X-22R, Beckman Coulter, Brea, CA, USA) at 4,500 *g* for 10 min at a temperature of 4°C. The pH of the liquid fractions, the hydrolysates, was adjusted to pH 2.0 with a 12 M solution of HCl to prevent unwanted microbial growth prior to experiments with *G. xylinus*. The hydrolysates were stored at 4°C until further use.

### Chemicals and microorganism

Activated charcoal (C2764), sodium hydroxide, calcium hydroxide, ammonium hydroxide solution, sodium sulfite, sodium dithionite, HMF, furfural, formic acid, acetic acid, vanillin, Folin-Ciocalteu reagent, catalase from *Aspergillus niger* and horseradish peroxidase were purchased from Sigma-Aldrich. Anion exchanger [AG 1-X8, 20–50 mesh, 3.2 meq/g (dry)] and cation exchanger [AG 50 W-X8, 20–50 mesh, 5.1 meq/g (dry)] were obtained from Bio-Rad (Richmond, California). Laccase from *Trametes (Coriolus) versicolor* was obtained from Jülich Fine Chemicals (Jülich, Germany).

The microorganism, *Gluconacetobacter xylinus* (formerly *Acetobacter xylinus*) ATCC 23770 was obtained from the American Type Culture Collection (Manassas, VA, USA). It was maintained on agar plates containing a seed culture medium consisting of 2.5% w/v glucose, 0.5% w/v yeast extract, 0.3% w/v peptone, and 2.0% w/v agar, and with an initial pH of 5.0.

### Detoxification of hydrolysate

Owing to the high concentration of inhibitors in the spruce enzymatic hydrolysate, it was diluted 3 times before use (one volume of hydrolysate was diluted with two volumes of Milli-Q water) and all concentrations reported refer to diluted hydrolysates. The detoxification treatment of spruce hydrolysate was performed in a 250 mL flask, which contained 120 mL diluted hydrolysate. The concentrations of monosaccharides (glucose, xylose, mannose, arabinose, and galactose), formic acid, acetic acid, furfural, HMF, and total phenols were measured before and after detoxification.

### Treatment with activated charcoal

Activated charcoal powder was added to the spruce hydrolysate to attain a 2% (w/v) suspension, which was then mixed vigorously. After 5 min at room temperature, the hydrolysate was centrifuged at 8300 *g* for 5 min. The supernatant was then adjusted to pH 5.0 with a 10 M aqueous solution of NaOH.

### Treatment with alkali

Alkali treatments of the spruce hydrolysate were performed (i) by adding NaOH to pH 9.0 and incubating for 3 h at 80°C, (ii) by adding Ca(OH)_2_ to pH 11.0 and incubating for 3 h at 30°C, or (iii) by adding 28% NH_4_OH to pH 9.0 and incubating for 3 h at 55°C. After the incubation, the hydrolysate was centrifuged at 8300 *g* for 5 min and the supernatant was then adjusted to pH 5.0 with a 10 M solution of H_2_SO_4_.

### Treatment with ion exchange resins

A strong anion-exchange resin, AG 1-X8, was used in its HO^–^ form. The cation exchanger AG 50 W-X8 (H^+^ form) was changed to sodium form by washing with a 1 M NaOH solution prior to use. The different resins were carefully washed with Milli-Q water several times and then the water was removed by filtration before use. In accordance with a previously described procedure [17], the ion exchangers were used in two different quantities. To 120 mL of the spruce hydrolysate, 6.2 g of the anion exchanger was added to reach pH 10.0, and 3.8 g was added to reach pH 5.5. The hydrolysate was stirred (120 rpm) with the anion exchanger for 1 h at room temperature (23°C) and was then filtered. For the cation exchanger, the corresponding amounts of ion exchange resin (6.2 g or 3.8 g) were first added to the hydrolysate, and then the pH was adjusted (to 10.0 for the 6.2 g suspension, and to 5.5 for the 3.8 g suspension) by addition of a 10 M solution of NaOH. The mixed suspension was stirred (120 rpm) at room temperature for 1 h and was then filtered. The pH of all filtrates was adjusted to 5.0 with a 10 M solution of H_2_SO_4_.

### Treatment with reducing agents

Two reducing agents, sodium sulfite and sodium dithionite, were used. Before the treatment, the pH of the spruce hydrolysate was adjusted to 5.0 with a 10 M solution of NaOH. The sulfur oxyanions, sulfite and dithionite, were added to the hydrolysate to a concentration of 10 mM. The mixture was stirred vigorously at room temperature for 15 min and was then filtered to remove insoluble material.

### Treatment with phenol-oxidizing enzymes

In a separate experimental series, two enzymatic detoxification methods were studied by using laccase from *Trametes versicolor* and peroxidase from horseradish. The specific activity of the laccase and the horseradish peroxidase was 2,600 and 50,300 units∙g^-1^ protein, respectively. Before enzymatic treatments, the pH of the hydrolysate was adjusted to 5.0 with a 10 M solution of NaOH. Portions of the different powdered enzyme preparations were dissolved in 100 mL pH-adjusted hydrolysate: (*i*) 88 mg (0.1 mg enzyme protein, 26 U) laccase, (*ii*) 440 mg (0.5 mg enzyme protein, 130 U) laccase, (*iii*) 11.8 mg (503 U) horseradish peroxidase, and (*iv*) 59 mg (2515 U) horseradish peroxidase. Controls for laccase and horseradish peroxidase were included and were treated in the corresponding way, except that they did not contain any enzyme preparation. All samples were incubated at 30°C for 12 h in a rotary shaker (90 rpm). One mL of a 20 mM solution of hydrogen peroxide was added once every hour to the samples containing horseradish peroxidase and to the corresponding control. At the end of the experiment with horseradish peroxidase, 4.5 mg of catalase from *Aspergillus niger* was also added to the reaction mixture [36]. To dilute the laccase reactions to the same extent as the horseradish peroxidase reactions, 12 mL of Milli-Q water was added to compensate for the hydrogen peroxide additions.

### Cultivation of *G. xylinus*

Cultivations of *G. xylinus* were performed to evaluate the effectiveness of the detoxification treatments. For comparison, untreated hydrolysate (i.e. control hydrolysate) and a reference medium containing five sugars (glucose, xylose, mannose, arabinose and galactose) in the same concentration as in untreated hydrolysate were included in the experiments. The cultivations were performed in triplicates. Production of bacterial cellulose was performed using *G. xylinus* ATCC 23770. In the first cultivation experiment, a series of 100-mL flasks were filled with 30 mL detoxified spruce hydrolysates, untreated hydrolysate, or reference medium consisting of 30 mL Milli-Q water and sugars (20 g/L glucose, 3.7 g/L xylose, 4.3 g/L mannose, 1.0 g/L galactose, and 0.7 g/L arabinose). In the second cultivation experiment, a series of 100-mL flasks were filled with 30 mL enzyme-treated spruce hydrolysate, control hydrolysate, or reference medium containing the same amount of sugars as in the control hydrolysate (18.7 g/L glucose, 3.0 g/L xylose, 3.3 g/L mannose, 0.5 g/L galactose and 0.5 g/L arabinose). All culture media were supplemented with 5 g/L yeast extract and 3 g/L tryptone and the initial pH was adjusted to 5.0. The flasks were autoclaved at 110°C for 30 min. The flasks were inoculated with 8% (v/v) *G. xylinus* inoculum (2.4 mL), which was pre-grown for 24 h in a synthetic medium (25 g/L D-glucose, 5 g/L yeast extract, and 3 g/L tryptone, pH 5.0). The flasks were incubated statically at 30°C for 12–14 days. During the cultivation the pH value, the cell viability, and the consumption of glucose were measured every two days. After cultivation, the BC was collected to calculate the yield. The concentrations of glucose, xylose, mannose, arabinose, galactose, formic acid, acetic acid, furfural, and HMF were determined.

### Analyses

#### Analysis of cell viability

Fluorescence was used to determine the fraction of living bacteria. Measurements were performed using the Live/Dead® Baclight^TM^ Bacterial Viability Kit (Invitrogen, NY, USA) and a 1420 Multilabel Counter (Perkin Elmer, Waltham, MA, USA).

#### Analysis of BC yield

The BC pellicles were collected after incubation and were washed thoroughly with distilled water. The weight of the BC pellicle was determined directly after drying at 105 ± 0.5°C for 24 h. The dried BC samples were weighed and the values were reported as g/L of the original medium.

#### Analysis of sugars

Concentrations of glucose, xylose, mannose, arabinose and galactose were determined by using high-performance anion-exchange chromatography (HPAEC). Before analysis, all samples were filtered through a 0.20 μm syringe-driven filter unit (Millex-GN, Millipore, Ireland) and diluted with Milli-Q water. The analytical system used was an ICS-3000 from Dionex (Sunnyvale, CA, USA) with an electrochemical detector. The separation was performed with a CarboPac PA20 (3 × 150 mm) separation column equipped with a CarboPac PA20 (3 × 30 mm) guard column (Dionex). Elution was performed with a 2 mM solution of NaOH during 25 min, followed by regeneration at 5 min with 100 mM NaOH, and equilibration for 15 min with 2 mM NaOH (Sodium Hydroxide Solution for IC, Sigma-Aldrich). The flow rate was 0.4 mL/min. Every two days during the cultivation, the consumption of glucose was monitored by using a glucometer (Glucometer Elite XL, Bayer Healthcare, Leverkusen, Germany).

#### Analysis of aliphatic acids

The concentrations of formic acid and acetic acid were determined with HPAEC by using the ICS-3000 system and its conductivity detector. All samples were filtered through the Millex-GN 0.20 μm syringe-driven filter unit, and diluted with Milli-Q water before analysis. The separation was performed with an IonPac AS15 (4 × 250 mm) separation column equipped with an IonPac AG15 (4 × 50 mm) guard column (Dionex). The mobile phase consisted of a 35 mM solution of NaOH (Sodium Hydroxide Solution for IC, Sigma-Aldrich), and the flow rate was 1.2 mL/min. External calibration curves were used for the quantification.

#### Analysis of furan aldehydes

The concentrations of the furan aldehydes HMF and furfural were determined by using high performance liquid chromatography (HPLC). A 1200 series instrument equipped with a UV G1315D detector was used (Agilent, USA). The eluent consisted of a mixture of Milli-Q water and acetonitrile, both of which contained 0.016% (v/v) trifluoroacetic acid (TFA). The gradient elution consisted of four steps: (*i*) 5% acetonitrile for 3 min, (*ii*) a linear increase to 100% acetonitrile after 13 min, (*iii*) a linear decrease to 5% acetonitrile after 18 min, and finally (*iv*) 5% acetonitrile until 23 min. The separation was carried out by using an ACE 5 C18-AR column (15 × 2.1 mm) (ACE, UK). Before analysis, all samples were filtered through the Millex-GN 0.20 μm syringe-driven filter unit and diluted with Milli-Q water.

#### Analysis of total phenols

The total phenolics were estimated using a Folin–Ciocalteu assay. Vanillin was used as a standard. For this assay, 0.2 mL of the diluted sample was mixed with 6 mL of Milli-Q water and 0.6 mL of Folin–Ciocalteu reagent (Sigma–Aldrich) and incubated at room temperature for 6 min. Then, 2 mL of 20% (w/v) Na_2_CO_3_ was added with mixing. After 120 min incubation at room temperature, the absorbance was measured at 760 nm by using a spectrophotometer (Shimadzu, Kyoto, Japan). The samples were filtered (0.2 μm pore size) before analysis.

## Competing interests

The authors declare that they have no competing interests.

## Authors’ contributions

All work has been carried out by XG and AC under the supervision of LJJ and FH. XG carried out the experiments, including the detoxification of spruce hydrolysate, the cultivation of *G. xylinus* and the analysis of sugars, furan aldehydes, and total phenols, analyzed the data and drafted the manuscript. AC prepared the spruce hydrolysate and determined the aliphatic acids with HPAEC. LJJ conceived the study, and revised the final manuscript. FH designed the study and helped to draft the manuscript. All authors read and approved the final version of the manuscript.

## Authors’ information

XG and AC are doctoral students with interests in the areas of enzymatic saccharification and bioconversion of biomass for the production of value-added products including biofuels and biopolymers. LJJ is a professor with focus on biotechnology for biorefining of lignocellulose. He is leader of the Biochemical Platform of the Bio4Energy research initiative (http://www.bio4energy.se). FH is a professor of biotechnology and bioengineering. His interests include low-cost production of bacterial cellulose and enzymes, bioconversion of renewable resources to high value-added products, as well as applications of biomaterials in biomedicine and functional materials.

## References

[B1] Gama M Gatenholm P Klemm D Bacterial nanocellulose: a sophisticated multifunctional material 2012 Boca Raton: CRC Press

[B2] Czaja WK Young DJ Kawecki M Brown RM The future prospects of microbial cellulose in biomedical applications Biomacromolecules 2007 8 1 12 10.1021/bm060620d 17206781

[B3] Jiang G Qiao J Hong F Application of phosphoric acid and phytic acid-doped bacterial cellulose as novel proton-conducting membranes to PEMFC Int J Hydrogen Energ 2012 37 9182 9192 10.1016/j.ijhydene.2012.02.195

[B4] Gao Q Shen X Lu X Regenerated bacterial cellulose fibers prepared by the NMMO · H_2_O process Carbohyd Polym 2011 83 1253 1256 10.1016/j.carbpol.2010.09.029

[B5] Hong F Zhu YX Yang G Yang XX Wheat straw acid hydrolysate as a potential cost-effective feedstock for production of bacterial cellulose J Chem Technol Biotechnol 2011 86 675 680 10.1002/jctb.2567

[B6] Chen L Hong F Yang XX Han SF Biotransformation of wheat straw to bacterial cellulose and its mechanism Bioresour Technol 2013 135 464 468 23186663 10.1016/j.biortech.2012.10.029

[B7] Hong F Qiu K An alternative carbon source from konjac powder for enhancing production of bacterial cellulose in static cultures by a model strain *Acetobacter aceti* subsp. *xylinus* ATCC 23770 Carbohyd Polym 2008 72 545 549 10.1016/j.carbpol.2007.09.015

[B8] Hong F Guo X Zhang S Han SF Yang G Jönsson LJ Bacterial cellulose production from cotton-based waste textiles: enzymatic saccharification enhanced by ionic liquid pretreatment Bioresour Technol 2012 104 503 508 22154745 10.1016/j.biortech.2011.11.028

[B9] Cavka A Guo X Tang SJ Winestrand S Jönsson LJ Hong F Production of bacterial cellulose and enzyme from waste fiber sludge Biotechnol Biofuels 2013 6 25 10.1186/1754-6834-6-25 23414733 PMC3610104

[B10] Skogsstatistisk Årsbok 2012 Jönköping: Skogsstyrelsen http://www.skogsstyrelsen.se/en/

[B11] Jönsson LJ Alriksson B Nilvebrant NO Bioconversion of lignocellulose: inhibitors and detoxification Biotechnol Biofuels 2013 6 16 10.1186/1754-6834-6-16 23356676 PMC3574029

[B12] Carvalheiro F Duarte LC Lopes S Parajó JC Pereira H Gírio FM Evaluation of the detoxification of brewery’s spent grain hydrolysate for xylitol production by *Debaryomyces hansenii* CCMI 941 Process Biochem 2005 40 1215 1223 10.1016/j.procbio.2004.04.015

[B13] Alriksson B Cavka A Jönsson LJ Improving the fermentability of enzymatic hydrolysates of lignocellulose through chemical in-situ detoxification with reducing agents Bioresour Technol 2011 102 1254 1263 10.1016/j.biortech.2010.08.037 20822900

[B14] Cavka A Alriksson B Ahnlund M Jönsson LJ Effect of sulfur oxyanions on lignocellulose-derived fermentation inhibitors Biotechnol Bioeng 2011 108 2592 2599 10.1002/bit.23244 21702027

[B15] Parajó J Domínguez H Domínguez J Xylitol production from *Eucalyptus* wood hydrolysates extracted with organic solvents Process Biochem 1997 32 599 604 10.1016/S0032-9592(97)00016-2

[B16] Larsson S Reimann A Nilvebrant NO Jönsson LJ Comparison of different methods for the detoxification of lignocellulose hydrolyzates of spruce Appl Biochem Biotechnol 1999 77 91 103 10.1385/ABAB:77:1-3:91

[B17] Nilvebrant NO Reimann A Larsson S Jönsson LJ Detoxification of lignocellulose hydrolysates with ion-exchange resins Appl Biochem Biotechnol 2001 91–93 35 49 11963864 10.1385/abab:91-93:1-9:35

[B18] Carvalho WD Canilha L Mussatto SI Dragone G Morales MLV Solenzal AIN Detoxification of sugarcane bagasse hemicellulosic hydrolysate with ion-exchange resins for xylitol production by calcium alginate-entrapped cells J Chem Technol Biotechnol 2004 79 863 868 10.1002/jctb.1061

[B19] Alriksson B Horváth IS Sjöde A Nilvebrant NO Jönsson LJ Ammonium hydroxide detoxification of spruce acid hydrolysates Appl Biochem Biotechnol 2005 121–124 911 922 15930570 10.1385/abab:124:1-3:0911

[B20] Alriksson B Sjöde A Nilvebrant NO Jönsson LJ Optimal conditions for alkaline detoxification of dilute-acid lignocellulose hydrolysates Appl Biochem Biotechnol 2006 130 599 611 10.1385/ABAB:130:1:599 16915672

[B21] Bruijn JM Kieboom APG Bekkum H Poel PW Reactions of monosaccharides in aqueous alkaline solutions Sugar Technol Rev 1986 13 21 52

[B22] Cantarella M Cantarella L Gallifuoco A Spera A Alfani F Comparison of different detoxification methods for steam-exploded poplar wood as a substrate for the bioproduction of ethanol in SHF and SSF Process Biochem 2004 39 1533 1542 10.1016/S0032-9592(03)00285-1

[B23] Chandel AK Kapoor RK Singh A Kuhad RC Detoxification of sugarcane bagasse hydrolysate improves ethanol production by *Candida shehatae* NCIM 3501 Bioresour Technol 2007 98 1947 1950 10.1016/j.biortech.2006.07.047 17011776

[B24] Intorne AC De Oliveira MVV Pereira LM De Souza Filho GA Essential role of the *czc* determinant for cadmium, cobalt and zinc resistance in *Gluconacetobacter diazotrophicus* PAI 5 Int Microbiol 2012 15 69 78 22847268 10.2436/20.1501.01.160

[B25] Sutherland JP Bayliss AJ Braxton DS Predictive modelling of growth of *Escherichia coli* O157:H7: the effects of temperature, pH and sodium chloride Int J Food Microbiol 1995 25 29 49 10.1016/0168-1605(94)00082-H 7599029

[B26] Nagata S Maekawa Y Ikeuchi T Wang YB Ishida A Effect of compatible solutes on the respiratory activity and growth of *Escherichia coli* K-12 under NaCl stress J Biosci Bioeng 2002 94 384 389 16233322

[B27] Wadskog I Adler L Hohmann S, Mager W Ion homeostasis in Saccharomyces cerevisiae under NaCl stress Yeast stress responses: volume 1 2003 Berlin Heidelberg: Springer 201 239

[B28] Martinez A Rodriguez ME Wells ML York SW Preston JF Ingram LO Detoxification of dilute acid hydrolysates of lignocellulose with Lime Biotechnol Prog 2001 17 287 293 10.1021/bp0001720 11312706

[B29] Dahman Y Jayasuriya K Kalis M Potential of biocellulose nanofibers production from agricultural renewable resources: preliminary study Applied Biochem Biotechnol 2010 162 1647 1659 10.1007/s12010-010-8946-8 20358409

[B30] Mikkelsen D Flanagan BM Dykes GA Gidley MJ Influence of different carbon sources on bacterial cellulose production by *Gluconacetobacter xylinus* strain ATCC 53524 J Appl Microbiol 2009 107 576 583 10.1111/j.1365-2672.2009.04226.x 19302295

[B31] Keshk S Sameshima K Evaluation of different carbon sources for bacterial cellulose production Afr J Biotechnol 2005 4 478 482

[B32] Taherzadeh MJ Gustafsson L Niklasson C Lidén G Conversion of furfural in aerobic and anaerobic batch fermentation of glucose by *Saccharomyces cerevisiae* J Biosci Bioeng 1998 87 169 174 16232445 10.1016/s1389-1723(99)89007-0

[B33] Deppenmeier U The unique biochemistry of methanogenesis Progs Nucl Acid Res Mol Biol 2002 71 223 283 10.1016/s0079-6603(02)71045-3 12102556

[B34] Taherzadeh MJ Gustafsson L Niklasson C Lidén G Physiological effects of 5-hydroxymethylfurfural on *Saccharomyces cerevisiae* Appl Microbiol Biotechnol 2000 53 701 708 10.1007/s002530000328 10919330

[B35] Vandamme EJ De Baets S Vanbaelen A Joris K De Wulf P Improved production of bacterial cellulose and its application potential Polym Degrad Stabil 1998 59 93 99 10.1016/S0141-3910(97)00185-7

[B36] Jönsson LJ Palmqvist E Nilvebrant NO Hahn-Hägerdal B Detoxification of wood hydrolysates with laccase and peroxidase from the white-rot fungus *Trametes versicolor* Appl Microbiol Biotechnol 1998 49 691 697 10.1007/s002530051233

[B37] Delgenes JP Moletta R Navarro JM Effects of lignocellulose degradation products on ethanol fermentations of glucose and xylose by *Saccharomyces cerevisiae*, *Zymomonas mobilis*, *Pichia stipitis*, and *Candida shehatae* Enzyme Microb Technol 1996 19 220 225 10.1016/0141-0229(95)00237-5

[B38] Mussatto SI Roberto IC Alternatives for detoxification of diluted-acid lignocellulosic hydrolyzates for use in fermentative processes: a review Bioresour Technol 2004 93 1 10 10.1016/j.biortech.2003.10.005 14987714

[B39] Klinke HB Thomsen AB Ahring BK Inhibition of ethanol-producing yeast and bacteria by degradation products produced during pre-treatment of biomass Appl Microbiol Biotechnol 2004 66 10 26 10.1007/s00253-004-1642-2 15300416

[B40] Larsson S Quintana-Sáinz A Reimann A Nilvebrant N-O Jönsson LJ The influence of lignocellulose-derived aromatic compounds on oxygen-limited growth and ethanolic fermentation by *Saccharomyces cerevisiae* Appl Biochem Biotechnol 2000 84–86 617 632 10849822 10.1385/abab:84-86:1-9:617

